# Association between novel inflammatory markers and severity of acute cholangitis: a retrospective cohort study

**DOI:** 10.3389/fmed.2026.1898185

**Published:** 2026-07-28

**Authors:** Yunxiao Lyu, Bin Wang, Zhong Lyu

**Affiliations:** Affiliated Dongyang Hospital of Wenzhou Medical University, Dongyang, Zhejiang, China

**Keywords:** acute cholangitis, disease severity, inflammatory biomarker, neutrophil-to-albumin ratio, prognostic indicator

## Abstract

**Background:**

Accurate assessment of the severity of acute cholangitis (AC) is crucial for guiding timely and appropriate interventions.

**Methods:**

In this retrospective cohort study, 1,647 patients with confirmed AC who were admitted to a single tertiary center between January 2014 and December 2023 were included. The patients were classified as mild (*n* = 1,075), moderate (*n* = 366), or severe (*n* = 206) on the basis of the Tokyo Guidelines 2018. Baseline values of the neutrophil percentage-to-albumin ratio (NPAR), blood urea nitrogen-to-albumin ratio, red blood cell distribution width-to-albumin ratio, and neutrophil-to-lymphocyte ratio were collected at admission.

**Results:**

All four markers were significantly higher in patients with more severe AC than in those with mild or moderate AC (*p* < 0.001 for all comparisons). The NPAR was significantly higher in patients with moderate or severe AC than in those with mild AC, with similar values in the moderate and severe groups (2.186 [mild], 2.876 [moderate], and 2.846 [severe]). The NPAR showed the highest area under the curve values, with 0.793 (95% CI, 0.770–0.815) for the mild vs. moderate-to-severe, 0.727 (95% CI, 0.693–0.762) for the mild-to-moderate vs. severe, and 0.815 (95% CI, 0.778–0.852) for the shock vs. non-shock groups. The optimal NPAR cut-off values were 2.513, 2.714, and 2.663, respectively. A restricted cubic spline analysis showed a significant overall association between these markers and the severity of AC (*p* < 0.001).

**Conclusion:**

The NPAR shows superior prognostic performance to the blood urea nitrogen-to-albumin ratio, red blood cell distribution width-to-albumin ratio, and neutrophil-to-lymphocyte ratio in predicting the severity of AC.

## Background

Acute cholangitis (AC) remains a common endoscopic and surgical emergency. The severity of AC ranges from mild to life-threatening conditions, such as severe AC or septic shock, necessitating precise risk stratification to guide timely interventions (1). An accurate assessment of the severity of AC is critical for optimizing treatment strategies, such as conservative management, endoscopic biliary drainage (primarily via endoscopic retrograde cholangiopancreatography [ERCP]), percutaneous drainage, or urgent surgical intervention (2). Traditional inflammatory markers, such as the white blood cell count and C-reactive protein concentrations, are widely used for assessing AC (3). However, the predictive power of these markers for the severity of AC is often limited because of their lack of specificity and sensitivity across diverse patient populations. This situation has prompted the investigation of novel biomarkers that may offer improved prognostic accuracy of determining the severity of AC.

In recent years, composite inflammatory indices, which are derived from routine laboratory parameters, have gained attention for their potential to enhance risk assessment in various inflammatory conditions (4–6). Among these, the neutrophil percentage-to-albumin ratio (NPAR), which integrates the neutrophil percentage (marker of systemic inflammation) with albumin (reflects nutritional status and liver function), has emerged as a promising indicator (7). While the NPAR has been investigated in contexts, such as sepsis (8), cardiovascular disease (9), and bacterial peritonitis (8), its application in assessing the severity of AC remains unclear.

These four indices were selected on the basis of three pre-specified criteria. First, all four share a composite immune-metabolic structure that couples a marker of systemic inflammatory activity (neutrophil percentage, the neutrophil-to-lymphocyte relationship, or red-cell anisocytosis) with a marker of host physiological and nutritional reserve (albumin) or immune exhaustion (lymphocytes), mirroring the dual pathophysiology of AC, in which biliary obstruction drives a systemic inflammatory response while hepatic and nutritional reserve is progressively depleted. Second, each index is computable from a single routine admission panel (complete blood count plus basic biochemistry), incurring no additional cost and remaining feasible in resource-limited settings. Third, the NPAR was chosen as the primary index precisely because, unlike the BAR, RAR, and NLR, it had not previously been investigated in AC, whereas the latter three provided established comparators with prior evidence in biliary and abdominal sepsis. We acknowledge that other immune-metabolic indices exist, such as the platelet-to-lymphocyte ratio (PLR) and the systemic immune-inflammation index (SII); evaluation of these additional composite indices represents a direction for future work.

To address this knowledge gap, this study evaluated the NPAR, along with other novel indices, namely the blood urea nitrogen-to-albumin ratio (BAR), red blood cell distribution width-to-albumin ratio (RAR), and neutrophil-to-lymphocyte ratio (NLR), for predicting the severity of AC. In a large cohort, we aimed to determine the prognostic utility of these markers, with a particular focus on establishing the NPAR as a novel tool for risk stratification in AC.

## Methods

### Study design and population

This retrospective cohort study included 1,647 patients diagnosed with AC at Dongyang People’s Hospital between January 2014 and December 2023, and the protocol was approved by the institutional ethics committee. Diagnosis and severity grading followed the Tokyo Guidelines 2018 (TG18) (1): a confirmed diagnosis required at least one criterion from each of the three TG18 domains—systemic inflammation (fever and/or shaking chills with supportive laboratory findings), cholestasis (jaundice with supportive laboratory data), and imaging evidence (biliary dilatation or an obstructive etiology)—and severity was graded as mild, moderate, or severe. Septic shock was defined according to the Sepsis-3 criteria as the persistent requirement for vasopressor therapy to maintain a mean arterial pressure (MAP) ≥ 65 mmHg together with a serum lactate concentration >2 mmol/L despite adequate fluid resuscitation (10). Patients were eligible if they were aged ≥18 years, were experiencing a first-time episode of AC, and had complete admission laboratory data obtained before antibiotic administration. Patients were excluded for a prior documented episode of AC (recurrent disease), a concurrent infectious or inflammatory disease unrelated to cholangitis, active malignancy receiving chemotherapy, or end-stage liver disease. Because this was a retrospective study, conditions that can influence RDW independently of AC—iron, vitamin B12, or folate deficiency, recent blood transfusion, and hemolysis—were neither pre-specified exclusion criteria nor systematically recorded, which we acknowledge as a limitation in the Discussion. Demographics, admission laboratory results, imaging findings, and clinical outcomes were retrieved from electronic medical records, and additional variables used to grade severity included the use of vasopressors (dopamine or norepinephrine), altered mental status, the PaO_2_/FiO_2_ ratio, serum creatinine, the international normalized ratio, and albumin.

The primary objective of this study was to evaluate the prognostic utility of the NPAR in predicting the severity of AC on the basis of the TG18 criteria. The secondary objective was to assess the prognostic performance of the BAR, RAR, and NLR in predicting the severity of AC.

### Data extraction

In our study, all laboratory parameters were consistently collected before the initiation of antibiotic therapy. Extracted variables included demographics (e.g., age and sex) and comorbidities. Additional data included vital signs (e.g., heart rate and blood pressure) and laboratory parameters (e.g., blood urea nitrogen, albumin, red blood cell distribution width [RDW], platelet count, neutrophil count, and lymphocyte count). Derived ratios were computed as follows: BAR = blood urea nitrogen (mg/dL) ÷ albumin (g/dL); RAR = RDW (%) ÷ albumin (g/dL); and NLR = neutrophil count ÷ lymphocyte count; and NPAR = neutrophil percentage (%) ÷ albumin (g/dL).

### Statistical analysis

Data are presented as the mean ± standard deviation for approximately normally distributed variables or the median with interquartile range (IQR) for non-normally distributed variables. All statistical tests were two-sided, with significance established at *p* < 0.05. Nonparametric variables were analyzed using the Mann–Whitney *U* test (two groups) or the Kruskal–Wallis test (across the three severity groups), while categorical variables were compared using the *χ*^2^ test or Fisher’s exact test, as applicable. An analysis of variance was used to evaluate trends in mean values across severity categories. A receiver operating characteristic curve analysis assessed the diagnostic accuracy of biomarkers, with optimal cut-offs determined by the Youden index. Restricted cubic spline (RCS) regression within logistic regression models was used to investigate potential nonlinear relationships between predictors and the severity of AC in patients. In each RCS plot, the *x*-axis is the marker value and the *y*-axis is the estimated odds ratio (OR) for the outcome relative to a reference value; the solid line is the point estimate and the shaded band its 95% confidence interval, the horizontal line marks an OR of 1.0, the marker value at which the curve crosses this line indicates the risk-onset threshold, and the inflection point (knot) indicates where the slope of the dose–response relationship changes. Statistical significance was defined as *p* < 0.05. Analyses were performed using R (version 4.2.2), SPSS Statistics (version 26), and GraphPad Prism (version 8) to ensure a robust and comprehensive evaluation. Given the exploratory nature of this study, no correction for multiple comparisons was applied. All reported *p*-values are two-sided and should be interpreted as hypothesis-generating rather than confirmatory.

## Results

### Baseline characteristics and inflammatory markers

**Figure 1:**
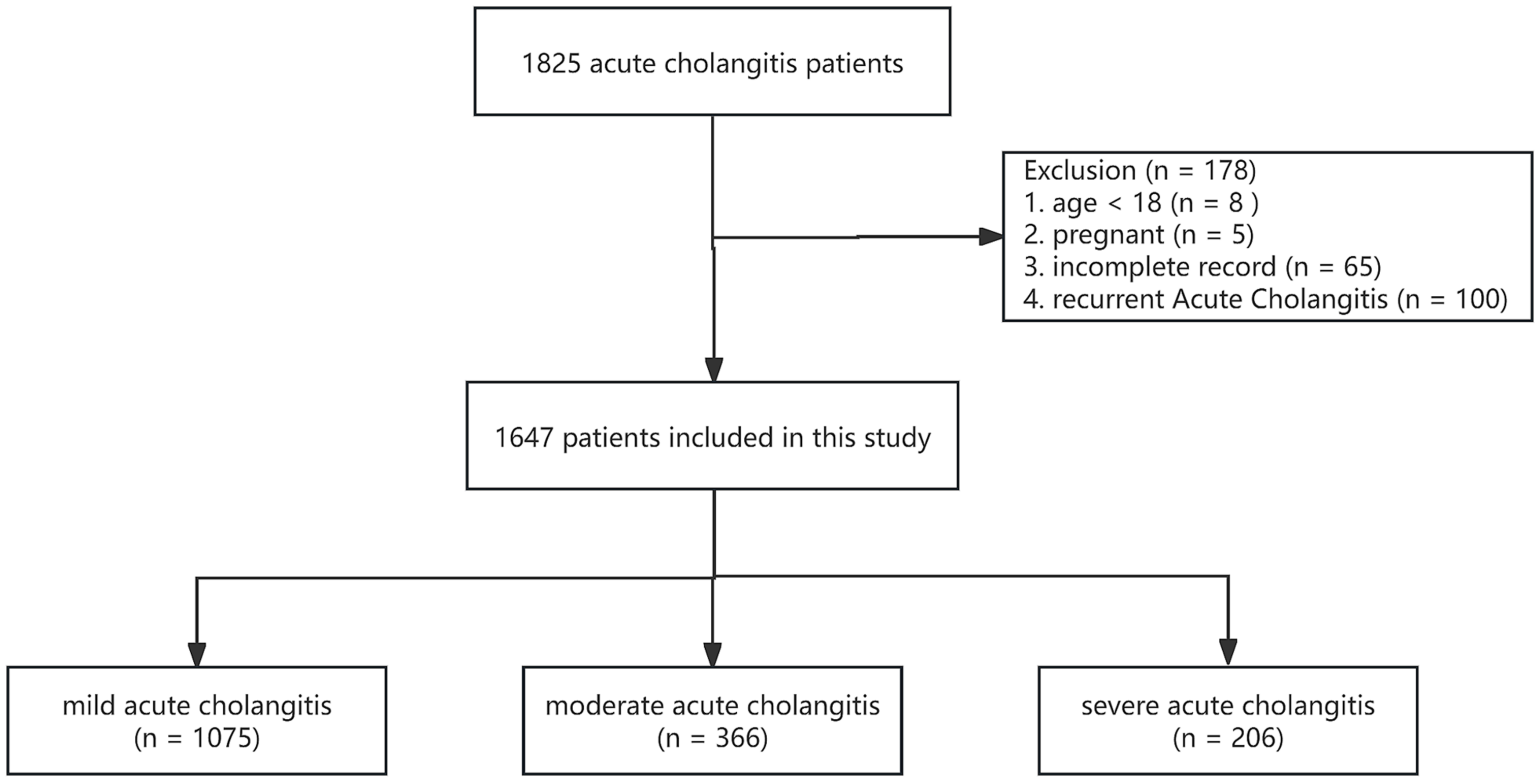
Patient screening flowchart.

This study included 1,647 patients with AC, which was classified according to the TG18 criteria as mild (*n* = 1,075 [65.3%]), moderate (*n* = 366 [22.2%]), or severe (*n* = 206 [12.5%]) (Figure 1). The patients’ baseline characteristics are shown in Table 1. Patients with moderate and severe AC were significantly older (mean [standard deviation] age, 75.18 [13.61] and 75.79 [12.88] years, respectively) than those with mild AC (62.60 [15.37] years) (*p* < 0.001). The sex distribution was not significantly different between the groups (*p* = 0.334). Laboratory findings are also shown in Table 1.

**Table 1:** Characteristics of included patients.

Characteristic	Mild (*n* = 1,075)	Moderate (*n* = 366)	Severe (*n* = 206)	*p* value
Sex				0.334
Male	595 (56.5)	199 (55.4)	125 (61.6)	
Female	458 (43.5)	160 (44.6)	78 (38.4)	
Age, y	62.60 ± 15.37	75.18 ± 13.61	75.79 ± 12.88	<0.001
Temperature, °C	36.99 ± 0.69	37.16 ± 0.85	37.22 ± 0.80	<0.001
WBC, ×10^9^/L	8.1 (6.0–10.7)	12.8 (8.8–15.5)	9.8 (7.0–14.1)	<0.001
Neutrophil, ×10^9^/L	6.5 (4.4–9.1)	11.4 (7.6–14.1)	8.7 (6.2–12.7)	<0.001
RDW, %	13.26 ± 1.50	13.92 ± 1.88	13.50 ± 1.47	<0.001
PLT, ×10^9^/L	193.12 ± 75.35	174.53 ± 84.69	159.97 ± 75.21	<0.001
TB, μmol/L	42.2 (22.8–71.3)	86.0 (37.4–123.6)	55.8 (33.3–89.0)	<0.001
ALB, g/L	37.06 ± 4.90	31.60 ± 6.07	31.88 ± 4.96	<0.001
AST, U/L	148.0 (65.0–296.5)	105.5 (52.2–186.0)	117.5 (57.5–215.8)	<0.001
CRE, μmol/L	62.0 (52.0–76.0)	66.0 (53.0–87.0)	74.0 (57.0–100.0)	<0.001
BUN, mmol/L	4.7 (3.6–6.3)	6.1 (4.4–8.1)	6.7 (5.1–9.5)	<0.001
CRP, mg/L	12.2 (2.7–50.9)	58.1 (16.3–118.5)	40.8 (10.0–95.2)	<0.001
PCT, ng/mL	0.7 (0.2–3.8)	2.9 (0.8–13.2)	3.3 (0.6–16.0)	<0.001
PT-INR	1.07 ± 0.16	1.21 ± 0.42	1.16 ± 0.20	<0.001
Cause				0.073
Choledocholithiasis	1,027 (95.5)	352 (96.2)	192 (93.2)	
Tumor	15 (1.4)	4 (1.1)	8 (3.9)	
Stent occlusion	5 (0.5)	3 (0.8)	3 (1.5)	
Other	28 (2.6)	7 (1.9)	3 (1.5)	
Therapy				<0.001
Pharmacological treatment	865 (80.5)	299 (81.7)	92 (44.7)	
Percutaneous biliary drainage	40 (3.7)	22 (6.0)	15 (7.3)	
Endoscopic treatment	159 (14.8)	85 (23.2)	94 (45.6)	
Surgical treatment	11 (1.0)	5 (1.4)	5 (2.4)	
Underlying disease				0.317
Hypertension	125 (11.6)	32 (8.7)	25 (12.1)	
Diabetes	85 (7.9)	15 (4.1)	12 (5.8)	
Coronary heart disease	78 (7.3)	21 (5.7)	19 (9.2)	
Chronic obstructive pulmonary disease	46 (4.3)	18 (4.9)	15 (7.3)	

Continuous variables are presented as mean ± standard deviation (SD) if approximately normally distributed, or as median (interquartile range, IQR) if non-normally distributed (WBC, neutrophil count, TB, AST, CRE, BUN, CRP, and PCT). Categorical variables are presented as number (percentage). *p* values were derived from one-way ANOVA (normally distributed continuous variables), the Kruskal–Wallis test (non-normally distributed continuous variables), or the *χ*^2^ test (categorical variables). Sex was available for 1,615 patients (32 missing). WBC, white blood cell count; RDW, red blood cell distribution width; PLT, platelet count; TB, total bilirubin; ALB, albumin; AST, aspartate aminotransferase; CRE, creatinine; BUN, blood urea nitrogen; CRP, C-reactive protein; PCT, procalcitonin; PT-INR, prothrombin time–international normalized ratio.

**Figure 2:**
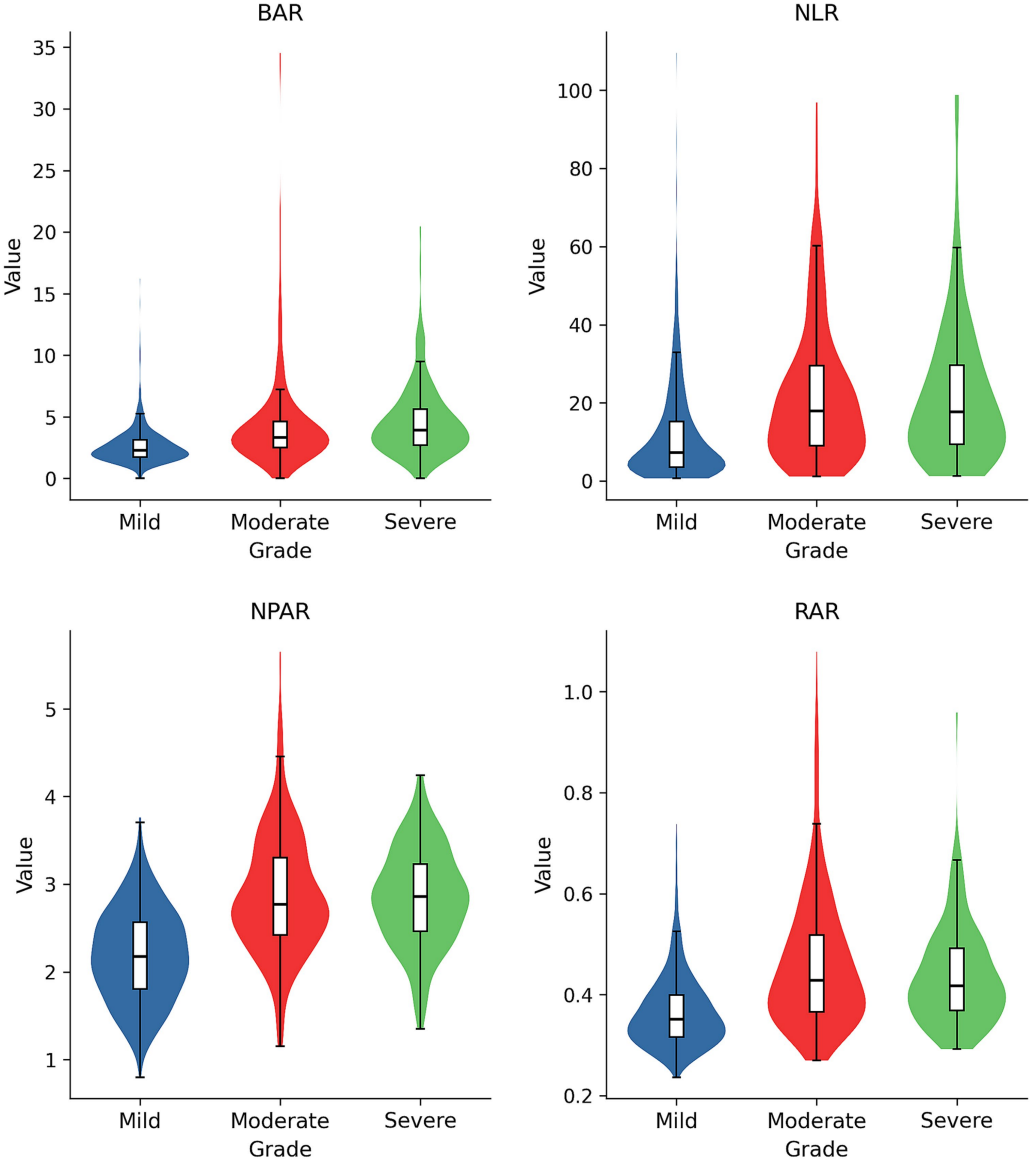
Distribution of inflammation markers across different severities of acute cholangitis. NPAR: neutrophil percentage-to-albumin ratio; BAR: blood urea nitrogen-to-albumin ratio; NLR: neutrophil-to-lymphocyte ratio; RAR: red blood cell distribution width-to-albumin ratio.

Table 2 shows the values of the inflammatory markers across the groups. The four markers differed significantly across the severity groups; the mean (standard deviation) values (severe vs. moderate vs. mild) were NPAR (2.846 [0.559] vs. 2.876 [0.691] vs. 2.186 [0.528]; *p* < 0.001), BAR (4.592 [2.87] vs. 4.31 [3.761] vs. 2.597 [1.449]; p < 0.001), RAR (0.436 [0.096] vs. 0.461 [0.132] vs. 0.365 [0.072]; p < 0.001), and NLR (22.117 [18.308] vs. 21.958 [17.497] vs. 11.64 [11.895]; p < 0.001) (Figure 2).

**Table 2:** NPAR, BAR, RAR, and NLR values across severity groups of acute cholangitis.

Variable	Mild (*n* = 1,075)	Moderate (*n* = 366)	Severe (*n* = 206)	*p* value
NPAR	2.186 (0.528)	2.876 (0.691)	2.846 (0.559)	<0.001
BAR	2.597 (1.449)	4.310 (3.761)	4.592 (2.870)	<0.001
RAR	0.365 (0.072)	0.461 (0.132)	0.436 (0.096)	<0.001
NLR	11.640 (11.895)	21.958 (17.497)	22.117 (18.308)	<0.001

Values are presented as mean (standard deviation). *p* values are for the overall difference across the three severity groups. NPAR, neutrophil percentage-to-albumin ratio; BAR, blood urea nitrogen-to-albumin ratio; RAR, red blood cell distribution width-to-albumin ratio; NLR, neutrophil-to-lymphocyte ratio.

### Prognostic associations of inflammatory markers

**Figure 3:**
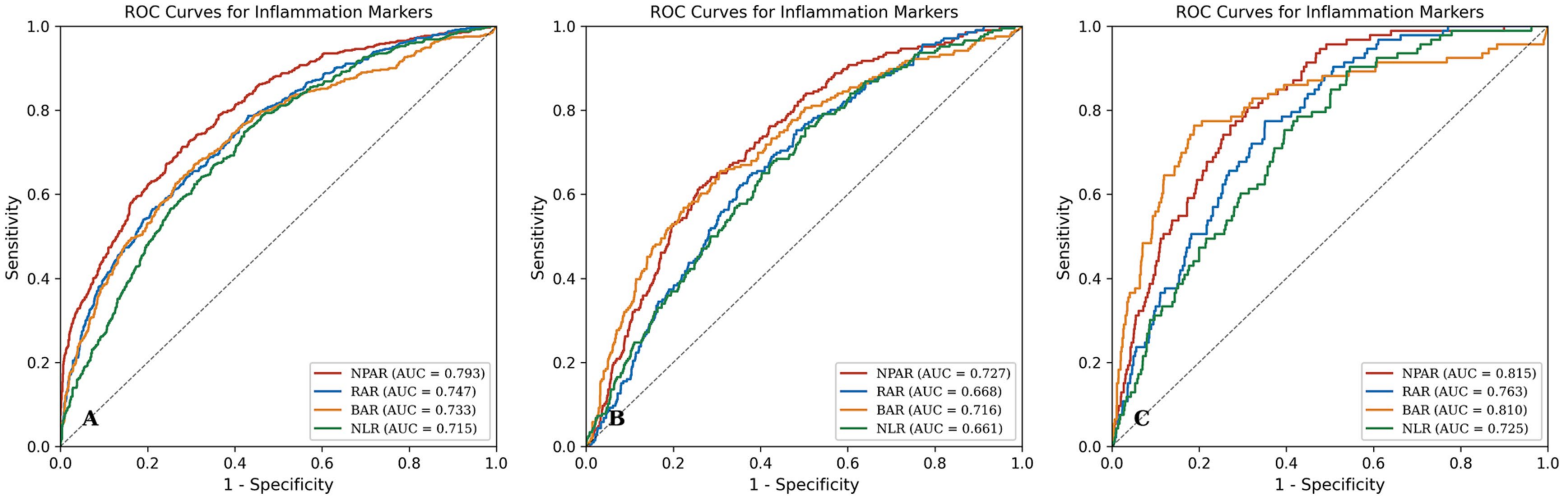
Receiver operating characteristic curves for inflammation markers in predicting the severity of acute cholangitis. **(A)** Mild vs. moderate-to-severe AC; **(B)** mild-to-moderate vs. severe AC; **(C)** shock vs. non-shock; NPAR: neutrophil percentage-to-albumin ratio; BAR, blood urea nitrogen-to-albumin ratio; NLR, neutrophil-to-lymphocyte ratio; RAR, red blood cell distribution width-to-albumin ratio.

Table 3 shows the associations between inflammatory markers and the severity of AC. The receiver operating characteristic curve analysis identified the NPAR as the most discriminative marker, with an area under the curve (AUC) of 0.793 (95% confidence interval [CI], 0.770–0.815) for mild vs. moderate-to-severe AC, using an optimal cut-off of 2.513 (Cohen’s *d*, 1.187) (Figure 3A). In mild-to-moderate vs. severe AC, the NPAR showed an AUC of 0.727 (95% CI, 0.693–0.762), with a cut-off of 2.714 (Cohen’s *d*, 0.760) (Figure 3B). In patients with shock vs. those without shock, the NPAR showed an AUC of 0.815 (95% CI, 0.778–0.852), with a cut-off of 2.663 (Cohen’s *d*, 1.198) (Figure 3C). The following other markers showed moderate performance for mild vs. moderate-to-severe AC. The BAR showed an AUC of 0.733 (95% CI, 0.707–0.759), with a cut-off of 2.882 (Cohen’s *d*, 0.771). The RAR showed an AUC of 0.747 (95% CI, 0.722–0.772), with a cut-off of 0.361 (Cohen’s *d*, 0.944). The NLR showed an AUC of 0.715 (95% CI, 0.689–0.740), with a cut-off of 9.292 (Cohen’s *d*, 0.730) (Figure 3).

**Table 3:** Association of inflammatory markers with the severity of acute cholangitis.

Variable	Group 1, mean (SD)	Group 2, mean (SD)	Cut-off	AUC (95% CI)	Cohen’s *d*
Mild vs. moderate-to-severe	Mild	Moderate-to-severe			
NPAR	2.186 (0.528)	2.863 (0.647)	2.513	0.793 (0.770–0.815)	1.187
BAR	2.597 (1.449)	4.411 (3.466)	2.882	0.733 (0.707–0.759)	0.771
RAR	0.365 (0.072)	0.452 (0.121)	0.361	0.747 (0.722–0.772)	0.944
NLR	11.640 (11.895)	22.015 (17.778)	9.292	0.715 (0.689–0.740)	0.730
Mild-to-moderate vs. severe	Mild-to-moderate	Severe			
NPAR	2.361 (0.647)	2.846 (0.559)	2.714	0.727 (0.693–0.762)	0.760
BAR	3.032 (2.389)	4.592 (2.870)	3.202	0.716 (0.677–0.755)	0.636
RAR	0.390 (0.100)	0.436 (0.096)	0.391	0.668 (0.632–0.704)	0.463
NLR	14.261 (14.259)	22.117 (18.308)	9.315	0.661 (0.624–0.699)	0.530
Shock vs. non-shock	Shock	Non-shock			
NPAR	3.137 (0.613)	2.379 (0.634)	2.663	0.815 (0.778–0.852)	1.198
BAR	6.320 (4.081)	3.042 (2.251)	3.898	0.810 (0.756–0.864)	1.371
RAR	0.480 (0.114)	0.390 (0.097)	0.399	0.763 (0.722–0.804)	0.911
NLR	25.783 (17.830)	14.612 (14.631)	12.000	0.725 (0.678–0.771)	0.753

For each marker and comparison, group values are reported as mean (standard deviation); the number in parentheses immediately after the mean is the SD. The cut-off is the optimal threshold determined by the Youden index. AUC, area under the receiver operating characteristic curve; the values in parentheses after the AUC are the 95% confidence interval (lower bound–upper bound). Cohen’s *d* is the standardized between-group effect size (absolute value). NPAR, neutrophil percentage-to-albumin ratio; BAR, blood urea nitrogen-to-albumin ratio; RAR, red blood cell distribution width-to-albumin ratio; NLR, neutrophil-to-lymphocyte ratio.

RCS analysis was performed to characterize the dose–response relationships between each inflammatory marker and AC severity across all three clinical comparisons. Overall associations were statistically significant for all markers (*P*_overall < 0.001).

### Mild vs. moderate-to-severe AC

**Figure 4:**
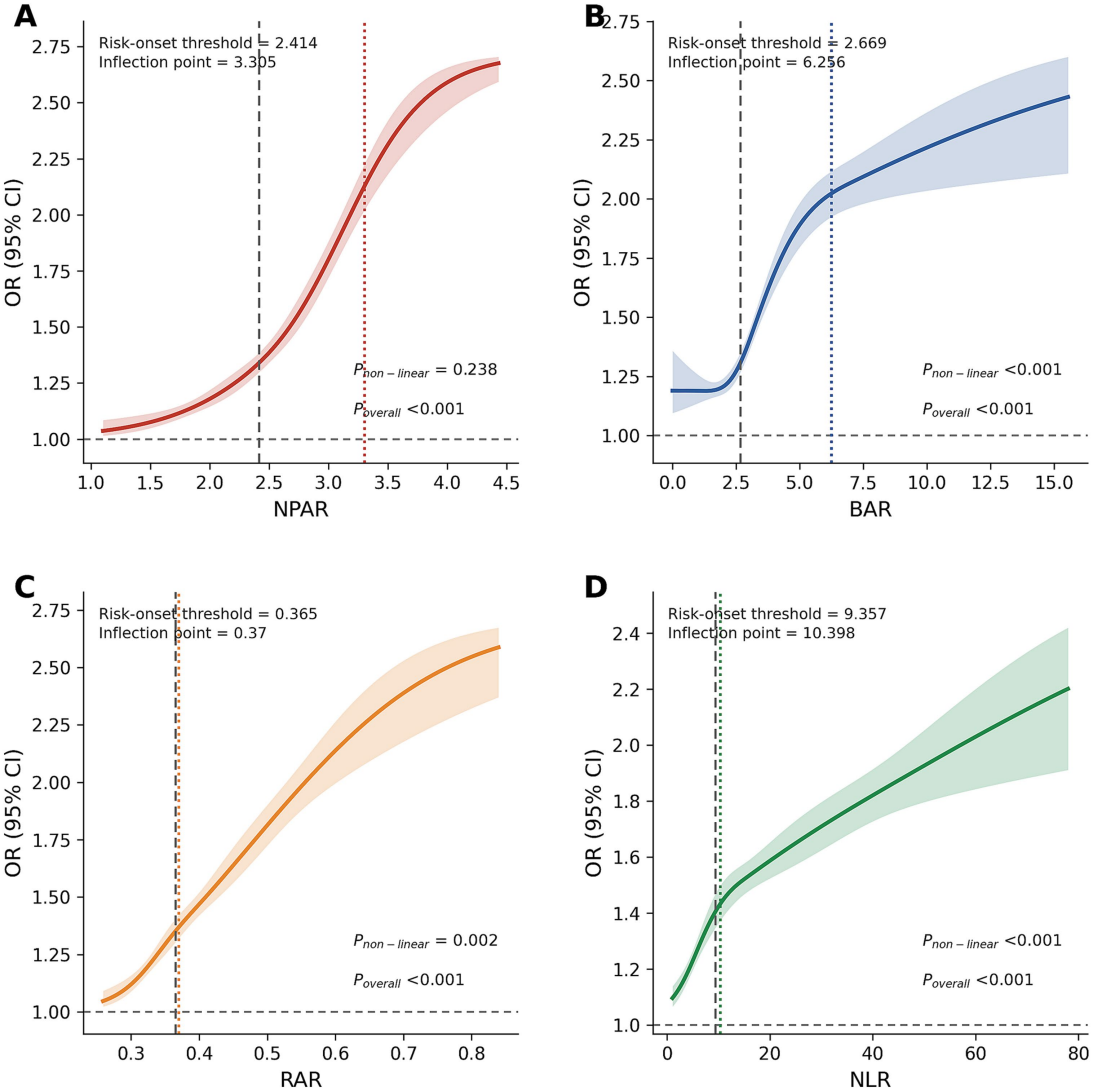
RCS of inflammatory markers for moderate-to-severe AC. **(A)**, NPAR; **(B)**, BAR; **(C)**, RAR; and **(D)** NLR. NPAR: neutrophil percentage-to-albumin ratio; BAR, blood urea nitrogen-to-albumin ratio; RAR, red blood cell distribution width-to-albumin ratio; NLR, neutrophil-to-lymphocyte ratio. In each panel, the *x*-axis is the marker value and the *y*-axis is the odds ratio (OR) relative to a reference value, with the shaded band showing the 95% confidence interval; the horizontal dashed line marks OR = 1, the grey dashed vertical line marks the risk-onset threshold (the marker value at which the OR crosses 1), and the coloured dotted vertical line marks the segmented-regression inflection point, with both values annotated on the panel. See the Statistical Analysis section for guidance on reading these curves.

For NPAR, the OR for moderate-to-severe disease began to exceed 1.0 at a value of 2.414, with segmented regression identifying a statistically significant inflection point at 3.305. The corresponding risk-onset thresholds and inflection points for the remaining markers were as follows: 2.669 and 6.256 for BAR; 0.365 and 0.370 for RAR; and 9.357 and 10.398 for NLR, respectively (Figure 4).

### Mild-to-moderate vs. severe AC

**Figure 5:**
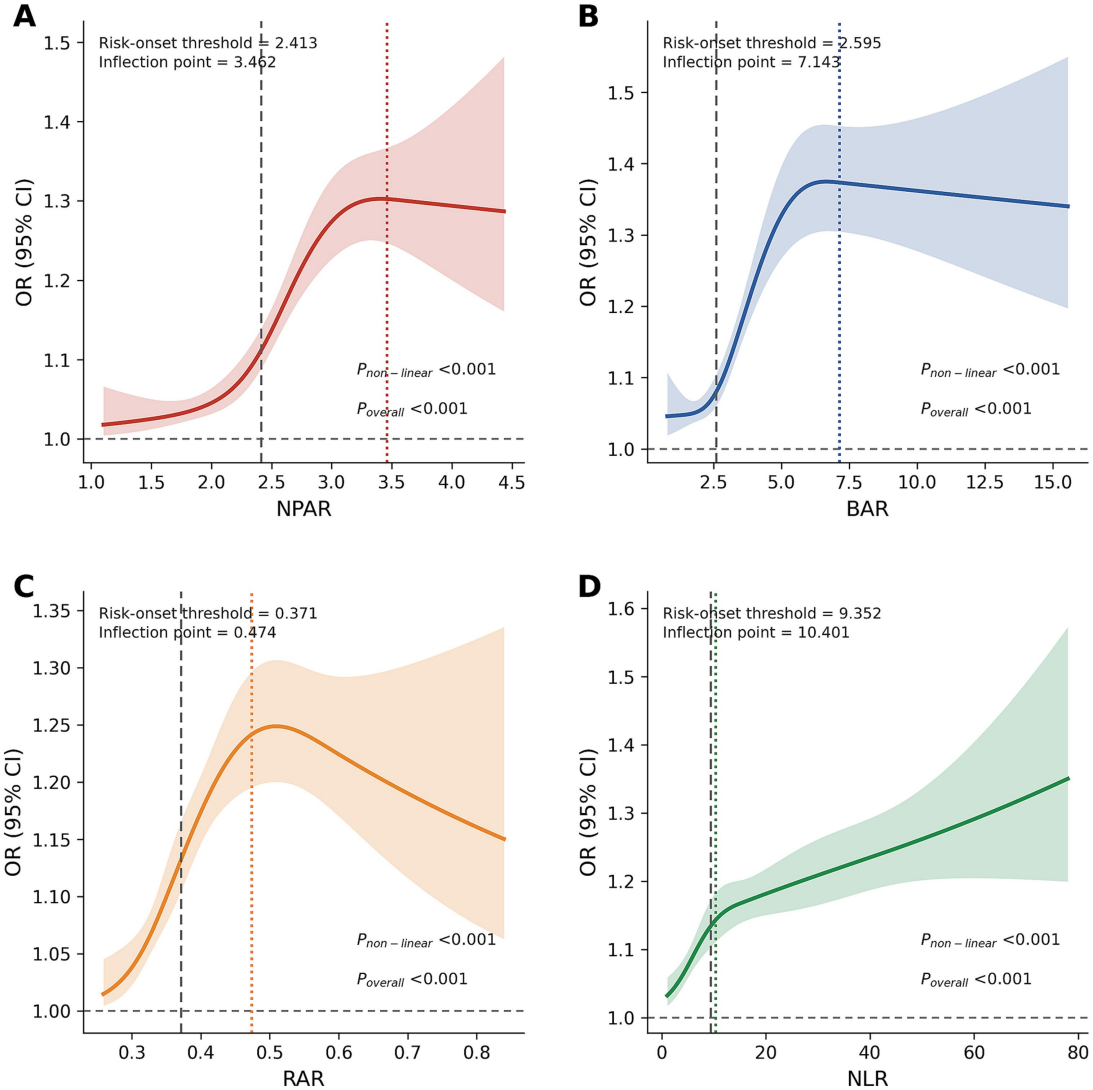
RCS of inflammatory markers for severe AC. **(A)**, NPAR; **(B)**, BAR; **(C)**, RAR; and **(D)** NLR. NPAR: neutrophil percentage-to-albumin ratio; BAR, blood urea nitrogen-to-albumin ratio; RAR, red blood cell distribution width-to-albumin ratio; NLR, neutrophil-to-lymphocyte ratio. In each panel, the *x*-axis is the marker value and the *y*-axis is the odds ratio (OR) relative to a reference value, with the shaded band showing the 95% confidence interval; the horizontal dashed line marks OR = 1, the grey dashed vertical line marks the risk-onset threshold (the marker value at which the OR crosses 1), and the coloured dotted vertical line marks the segmented-regression inflection point, with both values annotated on the panel. See the Statistical Analysis section for guidance on reading these curves.

Significant nonlinear associations were observed for all four markers (*P*_non-linear < 0.001 for all). The OR for NPAR exceeded 1.0 at a value of 2.413, with a segmented regression inflection point at 3.462. The risk-onset thresholds and inflection points for the remaining markers were: 2.595 and 7.143 for BAR; 0.371 and 0.474 for RAR; and 9.352 and 10.401 for NLR, respectively (Figure 5).

### Shock vs. non-shock

**Figure 6:**
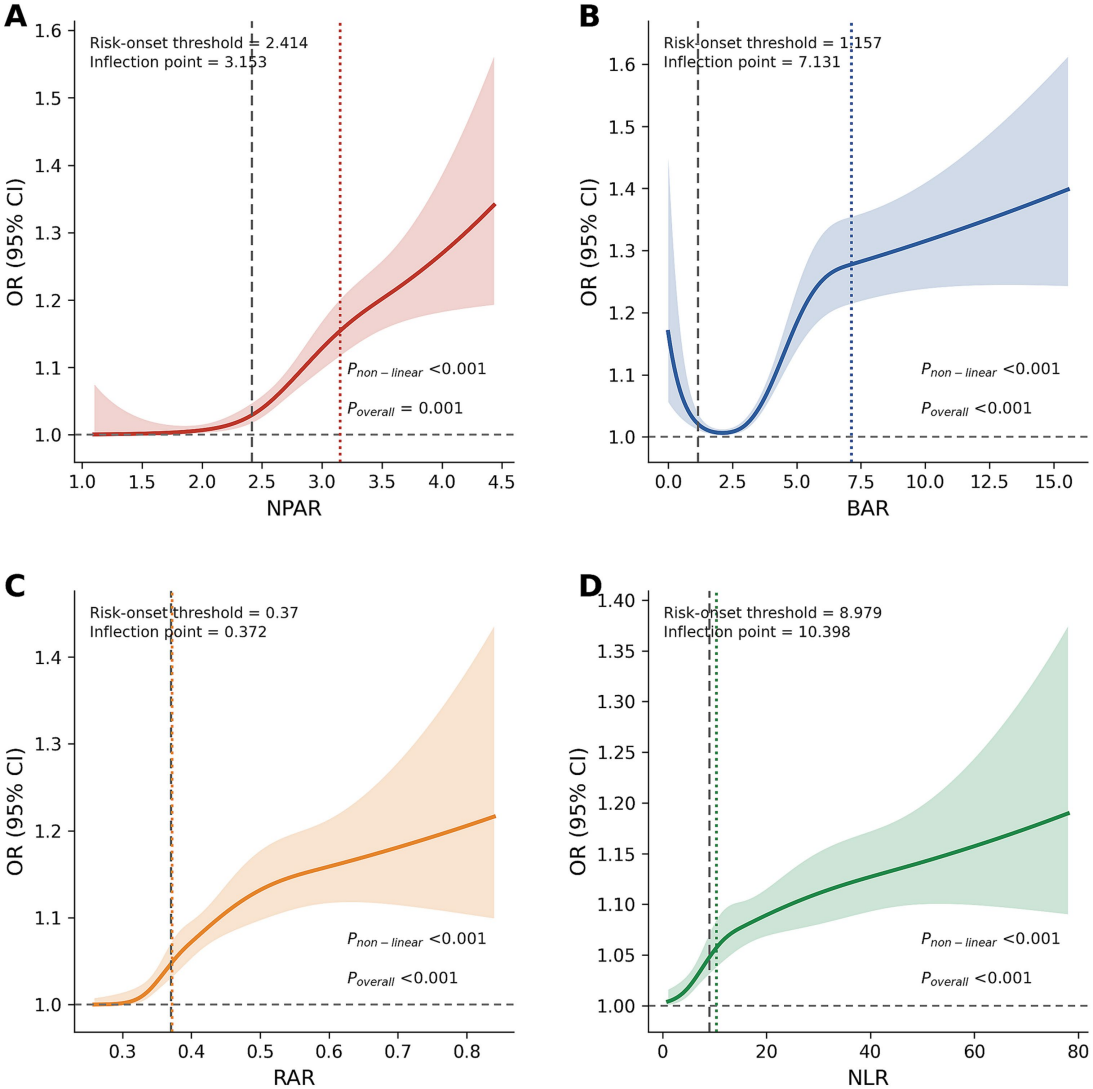
RCS of inflammatory markers for shock in AC. **(A)**, NPAR; **(B)**, BAR; **(C)**, RAR; and **(D)** NLR. NPAR: neutrophil percentage-to-albumin ratio; BAR, blood urea nitrogen-to-albumin ratio; RAR, red blood cell distribution width-to-albumin ratio; NLR, neutrophil-to-lymphocyte ratio. In each panel, the *x*-axis is the marker value and the *y*-axis is the odds ratio (OR) relative to a reference value, with the shaded band showing the 95% confidence interval; the horizontal dashed line marks OR = 1, the grey dashed vertical line marks the risk-onset threshold (the marker value at which the OR crosses 1), and the coloured dotted vertical line marks the segmented-regression inflection point, with both values annotated on the panel. See the Statistical Analysis section for guidance on reading these curves.

For NPAR, the OR began to exceed 1.0 at a value of 2.414, with an inflection point at 3.153 identified by segmented regression. The corresponding values for the remaining markers were: 1.157 and 7.131 for BAR; 0.370 and 0.372 for RAR; and 8.979 and 10.398 for NLR, respectively (Figure 6).

Detailed dose–response curves and marker-specific threshold values for all three comparisons are presented in Figures 4–6.

## Discussion

This study evaluated the prognostic utility of novel inflammatory markers, particularly the NPAR, in predicting the severity of AC using the TG18 criteria. We found that the NPAR was an effective biomarker, and it demonstrated superior discriminative ability to other indices, such as the BAR, RAR, and NLR. To the best of our knowledge, this is the first study to apply the NPAR for predicting the severity of AC. This finding could be helpful for risk stratification and clinical decision-making for emergency treatment.

### NPAR as a novel prognostic marker in AC

This study showed that the NPAR had the highest AUC values for mild vs. moderate-to-severe AC (0.793), mild-to-moderate vs. severe AC (0.727), and shock vs. non-shock (0.815). This finding highlights the potential of the NPAR as a potentially useful prognostic marker in patients with AC. Unlike traditional markers, such as the white blood cell count or C-reactive protein, which often lack specificity because of their broad inflammatory response (11), the NPAR integrates two physiologically relevant parameters. These parameters are the neutrophil percentage, which is a direct indicator of acute inflammatory activity, and albumin, which is a marker of nutritional status and hepatic synthetic function. This composite nature likely accounts for the enhanced sensitivity and specificity of the NPAR because it captures the inflammatory burden and the systemic physiological stress characteristic of severe AC.

The optimal cut-off values identified for the NPAR (2.513, 2.714, and 2.663 for the three severities) provide actionable thresholds for clinical use. These cut-offs, supported by substantial effect sizes (Cohen’s *d* ranged from 0.760 to 1.198), suggest that the NPAR can effectively differentiate patients at risk of progression to moderate-to-severe disease or septic shock. The RCS analysis further reinforced this finding, showing a significant overall association with a relatively stable increase in the OR across severity strata, despite a nonsignificant nonlinear trend in some comparisons (e.g., mild vs. moderate-to-severe). This stability enhances the reliability of the NPAR as a linear predictor, making it a practical addition to bedside assessment tools.

The novelty of applying the NPAR to the severity of AC lies in its prior validation in other systemic inflammatory states, such as sepsis (7), acute kidney injury (12), and acute myocardial infarction (9), where it has demonstrated prognostic value. Our study extends this framework to an intervention-relevant condition, bridging a critical gap in the literature (13). The prognostic utility of NPAR in AC is grounded in a coherent pathophysiological rationale. In AC, biliary obstruction elevates intraductal pressure, promoting bacterial translocation and endotoxemia that drives systemic neutrophil mobilization; markedly elevated neutrophil percentages thus serve as a direct indicator of inflammatory burden severity (14). Concurrently, on presentation the low serum albumin predominantly reflects redistribution into the interstitium due to increased capillary permeability, together with hemodilution from fluid resuscitation, rather than reduced hepatic synthesis. Because albumin has a half-life of approximately 21 days, suppression of synthesis cannot account for an acute decrease over the first hours to days of an episode; a genuinely low synthetic rate instead signals a pre-existing chronic process, such as chronic liver disease, malignancy, or chronic malnutrition (15). Hypoalbuminemia at admission therefore captures both the acute severity of the inflammatory insult, via redistribution and dilution, and any underlying chronic reserve deficit. By integrating these two complementary dimensions, NPAR captures both the intensity of the inflammatory insult and the host’s physiological reserve, offering more comprehensive prognostic information than either component in isolation (7).

### Comparative value of the BAR, RAR, and NLR

While the NPAR outperformed other markers, the BAR, RAR, and NLR were also significantly associated with the severity of AC, albeit with varying discriminative capacities. The BAR, with an AUC of 0.733 for mild vs. moderate-to-severe AC, reflects the interplay between renal stress (elevated blood urea nitrogen concentrations) and hepatic dysfunction (reduced albumin concentrations). The prognostic utility of the BAR may result from its frequent renal involvement in severe AC, particularly in patients progressing to systemic inflammatory response syndrome or multiorgan dysfunction. This finding is supported by the role of the BAR in predicting exacerbation of chronic obstructive pulmonary disease (16) and in AC-specific studies (17). However, the performance of the BAR was less consistent than that of the NPAR, with a sharper increase in the OR in the RCS analysis, suggesting a more threshold-dependent relationship that may limit its broad applicability.

The RAR, which combines the RDW—a marker of anisocytosis and inflammation—with albumin, showed an AUC of 0.747, indicating moderate predictive power. Mechanistically, the substrate of an elevated RAR in AC is twofold. Its numerator, RDW, reflects inflammation-driven dyserythropoiesis, in which pro-inflammatory cytokines (notably IL-6 and TNF-*α*) blunt the erythropoietin response, impair iron utilization, and shorten erythrocyte survival, thereby increasing anisocytosis; its denominator, albumin, falls acutely through capillary leak and dilution and, when chronically low, through reduced hepatic synthesis. Because RDW is a relatively slow-changing parameter, an elevated admission RAR may partly reflect a pre-existing chronic inflammatory or nutritional state rather than the acute insult alone. An elevated RDW is increasingly recognized as a surrogate for chronic inflammation and oxidative stress, which are prevalent conditions in severe AC (18). The significant nonlinearity of RAR in the RCS analysis suggests that the association between the RAR and severity of AC strengthens at higher values, potentially reflecting advanced disease states, such as acute pancreatitis (6) and post-coronary intervention mortality (18). These findings should not be directly extrapolated to acute cholangitis given the distinct pathophysiological mechanisms involved. The present study provides direct evidence supporting the discriminatory value of RAR specifically within the AC population.

The NLR, a well-established inflammatory index, achieved an AUC of 0.715, which is consistent with its widespread use in various inflammatory and oncological conditions. The elevation in the NLR in severe AC in our study is consistent with previous studies showing an association between neutrophilia and lymphopenia and poor outcomes in infectious diseases, such as Stevens–Johnson syndrome (11) and coronavirus disease 2019 (19). However, the lower AUC and smaller effect size (Cohen’s *d* = 0.730) of the NLR than the NPAR suggest that it may be less specific to the unique pathophysiology of AC, where local biliary inflammation and systemic sequelae coexist. The sharp increase in the OR of the NLR in the RCS analysis further indicates that its utility may be confined to a state of extreme severity, such as septic shock.

### Clinical implications and future directions

The integration of the NPAR into clinical practice could refine the TG18 framework, enabling earlier identification of patients requiring urgent intervention, such as endoscopic biliary drainage via ERCP, percutaneous cholecystostomy, or surgical intervention. The reliance of the NPAR on routine laboratory parameters (neutrophil percentage and albumin) ensures its feasibility in resource-limited settings, which is a considerable advantage over imaging-based methods or complex biomarker assays. Moreover, the complementary roles of the BAR, RAR, and NLR suggest a potential multi-marker approach, in which combining these indices could enhance prognostic accuracy, similar to scoring systems, such as APACHE II or SOFA in critical care.

Several limitations of this study warrant acknowledgment. First, the retrospective, single-center design introduces potential selection bias and precludes causal inference; prospective multicenter validation is required to confirm the generalizability of these findings across diverse clinical settings. Second, all analyses were based on single admission values of each marker; longitudinal assessment of NPAR dynamics may provide additional prognostic information regarding disease trajectory and treatment response. Third, comparative analyses with established inflammatory markers such as C-reactive protein or procalcitonin were not performed, and future studies should incorporate these comparisons to more comprehensively contextualize NPAR’s discriminatory performance (20). Fourth, the absence of comparison with validated prognostic scoring systems—including APACHE II and SOFA—represents a notable limitation attributable to incomplete data availability in this retrospective cohort; prospective studies should systematically incorporate these scores to formally evaluate the incremental value of NPAR. Fifth, an AUC of 0.727 and a Cohen’s *d* of 0.760 for the mild-to-moderate vs. severe comparison indicate moderate discriminatory accuracy, suggesting that NPAR is unlikely to be sufficient as a standalone risk stratification tool; its clinical utility is best realized in combination with other clinical and laboratory parameters or established scoring systems. Sixth, demographic and comorbidity variables—including BMI, history of kidney disease, and the Charlson Comorbidity Index—were incompletely documented due to the retrospective design and could not be incorporated into the analyses, which may further limit generalizability. In addition, the interval from symptom onset to hospital presentation could not be reliably determined, because the time of symptom onset was self-reported, frequently vague, and inconsistently documented; all laboratory values were therefore standardized to a fixed time point (admission, before antibiotic administration), which partially mitigates timing heterogeneity, and prospective collection of the onset-to-presentation interval is recommended for future studies. Furthermore, clinical conditions that affect RDW independently of AC—including iron, vitamin B12, or folate deficiency, recent blood transfusion, and hemolysis—were not pre-specified exclusion criteria and were not systematically recorded, so the RAR results should be interpreted as discriminatory associations rather than confounder-adjusted independent effects. Finally, given the exploratory nature of this study, no correction for multiple comparisons was applied; all findings should be interpreted with appropriate caution and require independent prospective validation.

## Conclusion

This study suggests that the NPAR is an important prognostic marker for the severity of AC, offering superior discriminative power over the BAR, RAR, and NLR. The application of the NPAR in this context heralds a meaningful addition to existing risk stratification frameworks, with the potential to optimize therapeutic decision-making and improve patients’ outcomes. While the BAR, RAR, and NLR provide useful adjunctive insights, NPAR’s simplicity, robustness, and pathophysiological relevance indicate that it could be important for future management strategies of AC. These findings will hopefully lead to larger-scale investigations to solidify the role of the NPAR in clinical hepatobiliary practice.

## Data Availability

The raw data supporting the conclusions of this article will be made available by the corresponding author upon reasonable request.
